# Development of the Tardivo Algorithm to Predict Amputation Risk of Diabetic Foot

**DOI:** 10.1371/journal.pone.0135707

**Published:** 2015-08-17

**Authors:** João Paulo Tardivo, Maurício S. Baptista, João Antonio Correa, Fernando Adami, Maria Aparecida Silva Pinhal

**Affiliations:** 1 Faculdade de Medicina do ABC, Santo Andre, SP, Brazil; 2 Center for the treatment of diabetic feet-Faculdade de Medicina do ABC, Santo Andre, SP, Brazil; 3 Universidade de São Paulo, Departamento de Bioquímica, Instituto de Quimica, São Paulo, SP, Brazil; Sapienza, University of Rome, School of Medicine and Psycology, ITALY

## Abstract

Diabetes is a chronic disease that affects almost 19% of the elderly population in Brazil and similar percentages around the world. Amputation of lower limbs in diabetic patients who present foot complications is a common occurrence with a significant reduction of life quality, and heavy costs on the health system. Unfortunately, there is no easy protocol to define the conditions that should be considered to proceed to amputation. The main objective of the present study is to create a simple prognostic score to evaluate the diabetic foot, which is called Tardivo Algorithm. Calculation of the score is based on three main factors: Wagner classification, signs of peripheral arterial disease (PAD), which is evaluated by using Peripheral Arterial Disease Classification, and the location of ulcers. The final score is obtained by multiplying the value of the individual factors. Patients with good peripheral vascularization received a value of 1, while clinical signs of ischemia received a value of 2 (PAD 2). Ulcer location was defined as forefoot, midfoot and hind foot. The conservative treatment used in patients with scores below 12 was based on a recently developed Photodynamic Therapy (PDT) protocol. 85.5% of these patients presented a good outcome and avoided amputation. The results showed that scores 12 or higher represented a significantly higher probability of amputation (Odds ratio and logistic regression-IC 95%, 12.2–1886.5). The Tardivo algorithm is a simple prognostic score for the diabetic foot, easily accessible by physicians. It helps to determine the amputation risk and the best treatment, whether it is conservative or surgical management.

## Introduction

Diabetes is a chronic disease, primarily related to insulin physiology but with consequences for the entire body. Around 10% of the American population suffer from diabetes and this percentage rises to 25% in the elderly [1].

In Brazil, 18.6% of the elderly population has been diagnosed as diabetic [2]. One of the major chronic complications of diabetes is associated with the foot, which can present various degrees of neurological and/or vascular disease and various degrees of ulceration, infection and necrosis with loss of tissue. Diabetic foot is the most common cause of non-traumatic amputations of lower limbs [3]. Approximately 80% of lower limb amputations are performed in patients with peripheral vascular disease and/or diabetes [2]. Amputation of the lower extremities is one of the most feared complications for patients with diabetes mellitus, causing significant reduction in mobility and quality of life.

Treatment of diabetic foot requires a multidisciplinary team to address the infection, swelling, pain, metabolic disorders, nutritional deficit, co-morbidities and surgical revascularization [4]. Several intensive-care strategies have been developed recently to avoid amputation, including the use of Photodynamic Therapy (PDT) protocols [5,6]. It is important to point out that for any strategy used to treat diabetic foot there is always the risk of amputation, which is still not well defined by the medical community. Understanding the chances of cure, aids in making the therapeutic decision and can reduce amputations and unnecessary costs related to hospitalization and prolonged treatment. The role of the physician in deciding amputation or clinical treatment is not easy [6,7]. The decision is frequently based on how bad the wound looks. However, this visual observation frequently hinders the decision-making process, because the appearances of a wound is definitely not a factor correlated with amputation.

Several authors have contributed with tools to identify risk factors and the odds of healing wounds of the diabetic foot. In 2006, Beckert and coworkers published a prospective study of one thousand patients, which created a score called DUSS (Diabetic Ulcer Severity Score) [7]. Each of the parameters is scored with a 0 or 1, and their sum will vary from 0 to 4. The higher the score, the worse the prognosis. However, patients with the same score can be classified into different subgroups with different prognoses, which may potentially confuse the clinician, complicating the final decision.

Barberan and coworkers developed an evaluation sheet containing ten items, with three ranks for each item, creating a record with thirty items to assess foot healing in type II diabetic patients [8]. The final score ranks the risk range for foot amputation, with the possibility of several outcomes. This score considers ten items such as location, topographical features, number of affected areas, ischemia, infection, edema, neuropathy, depth, area and phase of ulcer healing. Each item can be evaluated using three degrees, thus creating grades for each foot examined. Patients were stratified into stages I, II and III, grade III being the worst prognosis [8]. Despite the enhanced criteria, this classification did not infer a specific percentile for risk of amputation. It is also a bit complex to use, specially by less experienced physicians.

Lipsky developed and validated a risk score for amputation in hospitalized patients (over 3,000 patients) who presented infected diabetic foot. This study considered fourteen different factors associated with risk of amputation. The most significant factors analyzed were infection at the surgical site, vasculopathy, previous amputation and leukocytes over 11,000/mm^3^ [9]. The Lipsky score is a predictive, five-layered laminate system with scores ranging from 0 to 21 or more. However, there is no practical guidance in terms of defining how to use these scores to define the risk of amputation.

Although all these strategies have provided important information to evaluate the possible outcome of the diabetic foot, they are cumbersome and doctors tend to use empirical evaluations rather than performing the necessary quantifications. Therefore, new strategies must be developed to classify the diabetic foot in order to improve healing, speed up the healing process and avoid amputation, irrespective of the type of intervention or treatment.

The evidence to justify classifications of foot risk derives from a number of large cross-sectional and prospective studies and from the identification of clinical features in individual patients related to the relative risk of future ulceration. If foot risk classification is linked to preventive strategies, the incidence of new foot disease will fall. Therefore, foot risk classification should become a routine part of diabetes treatment [10]. A multidisciplinary approach together with a vascular surgeon is needed, since peripheral arterial occlusive disease and foot ulcers in diabetic patients increase the risk of foot complications and amputation [11].

As previously demonstrated Photodynamic Therapy (PDT) significantly improved the treatment of diabetic foot, avoiding amputation in around 94% of diabetic patients while also ameliorating osteomyelitis [5]. Therefore, we used PDT as the conservative treatment in order to evaluate the different parameters that are related with the progression and prognosis of the disease. Such parameters were used to create a score of classification for the diabetic foot. Although all patients of this study were treated with PDT, other efficient methods of conservative management of the diabetic foot could be applied, instead of PDT.

The present study aims to establish a simple prognostic score for the diabetic foot that can be quickly accessed by the physician allowing therapeutic decisions based on outcome probabilities. This score was based on the clinical practice of Dr. João Paulo Tardivo with its interdisciplinary team of health professionals in a public hospital. Our aim was to reduce the number of amputees, to improve patient quality of life and to reduce the social costs of this disease by employing this specific scoring strategy.

## Methods

### Patients

62 patients with diabetic foot, from March 2011 to March 2013 were used to develop the scoring method. The patients were treated at the Center for Diabetic Foot at Hospital Anchieta (CeDiFo), served by the Faculdade de Medicina do ABC (FMABC), which is coordinated by Dr. Tardivo. The study was approved by the research ethics committee at FMABC and participants signed a consent form. The clinical investigation was conducted according to the principles expressed in the Declaration of Helsinki.

Diabetic patients referred for amputation were submitted to PDT, and the following outcomes were observed: ulcer healing, osteomyelitis remission, referral to orthopedic foot specialist, referral for revascularization and surgery for amputation, as previously described [5,12].

In an effort to speed healing and fight infection Photodynamic therapy (PDT) was used in the patients. PDT has been proven to be an extremely efficient strategy to save the diabetic foot and it was always performed in an outpatient setting. In recent decades the worldwide rise in antibiotic resistance has driven research to develop new anti-microbial strategies using PDT. The use of photosensitizers such as phenothiazine, porphyrines, phthalocyanines and fullerenes have demonstrated antimicrobial efficacy against a broad spectrum of antibiotic resistant microorganisms [13]. Light irradiation of patients’ bones using optical fibers has been used and shown positive results for osteomyelitis [12,14].

PDT sessions were held twice a week in cases presenting excessive exudate, whether purulent or not, and once a week when the lesions presented less exudate, showing clinical signs of improvement. Feet were photographed every visit for a visual record of the progress of the wounds, in addition to maintenance of medical records. Patients who presented osteomyelitis had their feet x-rayed every 20 to 30 days to review the compromised bones. The number of PDT sessions and the time until outcome varied depending on the clinical presentation of each case.

Radiographic examination, blood count, C-reactive protein and erythrocyte sedimentation rate were performed to monitor infections. The photosensitizing agents used were phenothiazine methylene blue 1% and toluidine blue 1%. Prototype light sources were built using high energy LEDs (light emitting diode), emitting red light, or dichroic halogen lamp coupled with fibers optics. These prototypes have a power of 100 mW and emit red light between approximately 600 and 650 nm lambda. These protocols were previously used by Tardivo and coworkers and described [5,12].

Outcomes were defined as amputation or no amputation. Amputees or patients who did not heal were discharged from treatment or received other referrals, such as orthopedic assessment and intervention. Patients with signs of peripheral arterial disease were referred for angiography with angioplasty or revascularization whenever possible.

### Defining the score parameters

The practice outcome of these patients indicated that the chances of amputation, i.e. the prognosis score, could be based on three main factors: Wagner classification, signs of peripheral arterial disease; and location of the ulcer on the diabetic foot.

### Wagner Classification

**Fig 1:**
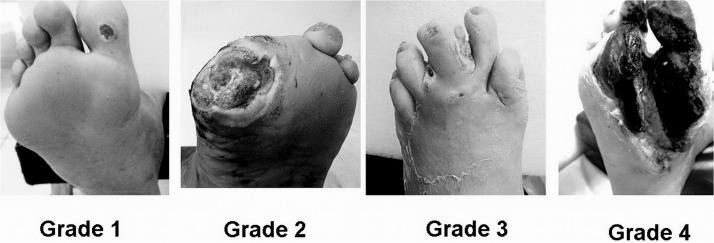
Wagner Classification for diabetic foot. Grade 1, superficial ulcer; Grade 2, Deep ulcer; Grade 3, osteitis-infection and Grade 4, forefoot gangrene.

Only patients with Wagner classification grades 1 to 4 were enrolled in the study. Fig 1 illustrates Wagner classification for the diabetic foot. It is important to point out that Wagner zero means no ulcer and Wagner 5 means the entire foot is gangrenous, neither case were clinically treated by PDT. For purposes of the prognosis, patients assessed as Wagner 1 through 4 were given a coinciding score, that is, 1, 2, 3 and 4.

### Peripheral Arterial Disease Classification

Characterization of peripheral arterial disease (PAD) was obtained from the *Peripheral Arterial Disease Classification*, which considers pallor of the extremities, no palpable distal pulses, ankle brachial index under 0.7, no digital perfusion, fixed cyanosis and dry gangrene. Good peripheral perfusion received a score of 1 (PAD 1 classification), while clinical signs of ischemia received a score of 2 (PAD 2 classification).

### Ulcer Localization

**Fig 2:**
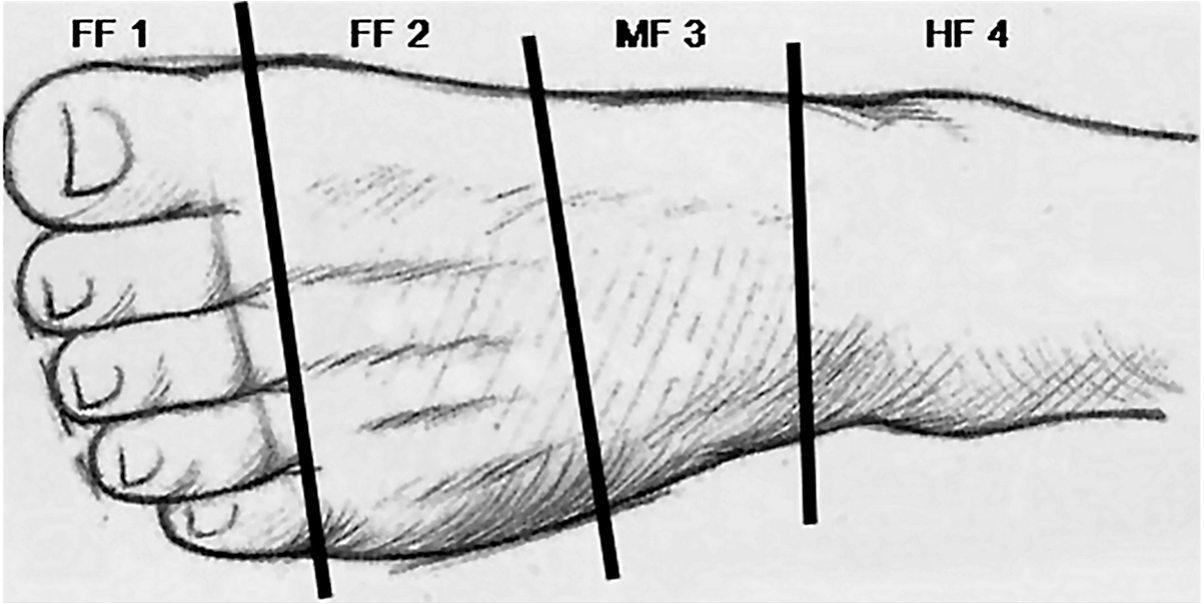
Location of foot injuries. FF, forefoot; MF, midfoot; HF, hindfoot. The localization sites showed at the figure are important to define the grades to calculate the algorithm.

Foot ulcers are defined according to their location, as shown in Fig 2. Forefoot 1 (FF1), region of the phalanges, forefoot 2 (FF2), region of the metatarsals, midfoot 3 (MF3), region covering the cuneiform, cuboid, and navicular bones, bounded by Lisfranc and Chopart joints, and hind foot 4 (HF4), heel area at the calcaneus and the talus. The score number follows the site of the ulcer, respectively, 1, 2, 3 or 4 and is independent of the depth or dorsal/plantar location.

### Final Score

The final score prediction is the product of these three scores: Wagner classification, presence of PAD and ulcer localization. The product of these three scores according to the variables mentioned above will result in a score of 1 to 32. The higher the score, the worse the prognosis for the diabetic foot.

## Results

### Clinical Features

Of the 62 patients enrolled in the study, 41 (66.1%) were male. The mean age of all patients was 58.7 years, median 58 years, with a minimum age of 32 and maximum of 81 years. Regular insulin was used by 71% of the patients. Previous amputation had occurred in 38 cases (61.3%). Major amputations had been performed on 7.9% of patients while small amputation had occurred in 92.1% of patients, 21.05% of which trans-metatarsal type amputation and 71.05% toe amputation. Table 1 shows outcomes of the patients after PDT treatment, as previously described. Amputations were avoided in the majority of cases (85.5%) of the patients submitted to conservative treatment using PDT (Table 1).

**Table 1:** Distribution of patients with diabetic foot and respective outcomes.

Outcome	N	%
Amputations	9	14.5
Clinical cure	40	64.5
Orthopedic Treatment	11	17.7
Referral for revascularization	2	3.3
Total	62	100

Wagner classification was crucial to identify the depth of the ulcer, the presence or absence of infection and the presence or absence of ischemic gangrene in ulcers ranged from small perforating plantar to a large stump in the healing phase.

The distribution of the patients according to Wagner classification was as follows: Wagner 3 (45 patients, 72.5%), Wagner 2 (9 patients, 14.5%), Wagner 4 (5 patients, 8.06%) and Wagner 1 (3 patients, 4.8%).

Table 2 shows that 64.3% of patients classified as PAD 2 underwent amputation while no case of amputation was observed in the patients classified as PAD 1, suggesting that PAD 2 is highly associated with amputation. Therefore, in the CeDiFo today´s practice, the first and most urgent concern is the care of the patient who presents PAD 2, due to the high risk of amputation. Angiographic evaluation followed by angioplasty or bypass grafts should be performed in this group of patients. After revascularization patient can return to conservative treatment using PDT.

**Table 2:** Distribution of patients with diabetic foot following PAD and amputation.

Peripheral Artherial Disease (PAD)	Surgical Procedure			
	Amputated		Not Amputated	
	N	%	N	%
PAD 1			48	100
PAD 2	9	64.3	5	35.7
Total	9	14.5	53	85.5

Ulcers were distributed as follows: forefoot 1, 13 patients (21.0%); forefoot 2, 28 patients (45.2%); midfoot, 12 patients (19.3%) and hind foot, 9 patients (14.5%). Table 3 presents the average healing time according to the location of the ulcer. The lesions located on the hind foot take an average of 234 days to reach outcome, while superior lesions located on the forefoot phalanges presented a faster outcome as shown in Table 3.

**Table 3:** Time (days) for clinical cure according to foot lesions localization.

Foot injury localization	Average of days for outcome
Forefoot 1 (phalanges)	80
Forefoot 2 (metatarsal)	118
Midfoot	123
Hind foot	234

**Fig 3:**
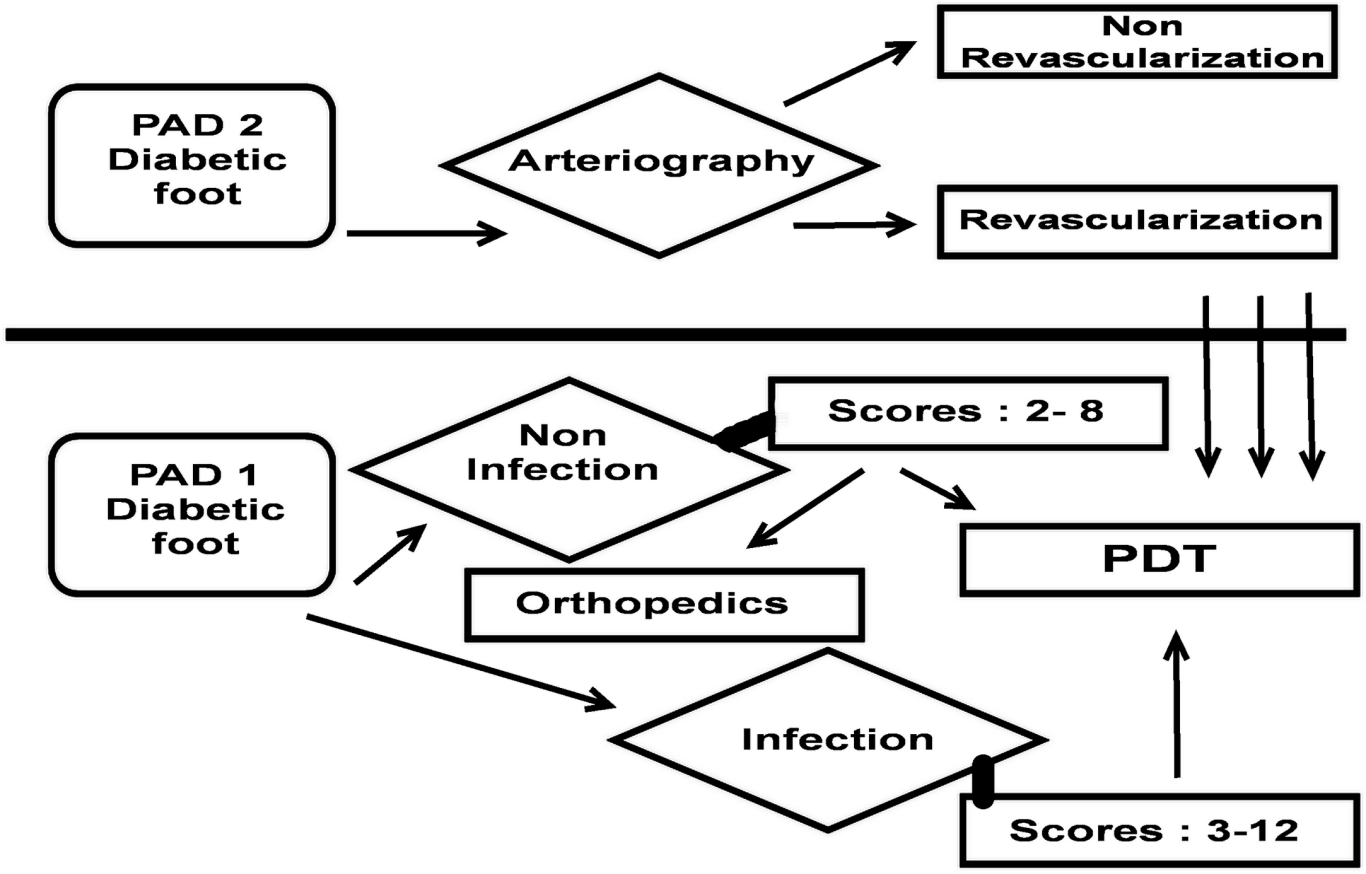
Strategic diagram of Tardivo Algorithm. The diagram indicates the parameters significantly important to evaluate diabetic foot and obtain the score values.

Fig 3 displays a flowchart of diabetic foot management defined by Tardivo. Scores above 12 present a high risk of amputation, whilst scores below 12 have a high chance of healing (Table 4). It was also observed that scores above 12 require PAD 2, consequently this parameter ends up serving as a threshold.

**Table 4:** Distribution of patients and outcomes according to obtained score.

Scores	Patients		Clinical Cure		Amputations	
	N	%	N	%	N	%
16 to 32	10	16.1	0	0	7	70
12	5	8.06	2	40	1	20
8 and 9	7	11.3	4	57.1	1	14.3
6	23	37.1	19	82.6	0	0
2 to 4	17	27.4	15	88.2	0	0

Table 4 shows the percentage of patients according to score and consequent outcome. It is clear that the higher frequency of amputation was obtained in the group of patients who received scores ranging from 16 to 32 (70.0%) and no amputations occurred with scores equal to or less than 6 (Table 4).

The Tardivo Algorithm was based on an evaluation of these 62 diabetic patients, who were subjected to a statistical analysis, by comparing the scores and the clinical outcomes. Logistic regression showed that for scores of 12 or higher the odds of amputation are 152 times higher than lower scores (IC 95%, 12.2–1886.5), as shown in Table 5.

**Table 5:** Odds ratio and logistic regression obtained by score groups.

Score	Odds Ratio (IC 95%)	p
0 to 11	1	<0.001
Equal or higher than 12	152 (12.2–1886.5)	

IC. confidence interval

## Discussion

Diabetes causes serious metabolic changes allowing localized infections to progress at a quicker rate. Diabetes and/or infection can also cause thrombosis in arteries, and rapid onset of ischemia that can compromise the vitality of all toe tissues, causing gangrene, which in turn requires amputation. It is very difficult to ensure an accurate prognosis for a diabetic foot. Therefore, a predictive score may be useful to predict and better define within a range of probability, and thereby reduce aggressive management in many cases. Greater emphasis should be placed on patient-related outcome parameters in order to compare the effectiveness of diabetic foot management in different health centers [11]. The sole evaluation of the extent of the ulcer underestimate the true morbidity and mortality associated with diabetic foot disease. Foot complications are really important when PAD is associated, significantly increasing amputation risk [15,16,17].

The Tardivo algorithm, which was based on the Wagner classification, the presence or absence of peripheral arterial disease and anatomic location of the ulcer, is a simple, quick and direct way to calculate a score and identify the risk of amputation in diabetic patients with foot complications. Therefore, according to the Tardivo algorithm, if a patient presents no ischemic alteration of the foot and scores below 12, this patient has a low probability of suffering some type of amputation, so we can take the risk and opt for a conservative treatment like PDT.

A multicenter study conducted in several European countries by the EURODIALE Study Group argues that the diabetic foot ulcer, with or without peripheral arterial disease (PAD), should be defined as two different states of illness [18]. This means that the presence of symptoms and/or signs of vascular disease worsen the prognosis. Similarly, the Multidisciplinary Group on the Diabetic Foot (Freital, Germany) argues that the presence of PAD hinders the very good result of the treatments. Therefore, there is previous evidence indicating that peripheral arterial disease should be considered a risk factor, which is in agreement with our results [19,20]. Another observation that we could glean from the clinical outcomes of diabetic patients with foot complications is that the location of the ulcers must also be considered a predictive factor. In fact, the data suggested a relationship between lesion location in the diabetic foot and the time for the denouement.

The ideal treatment of diabetic foot requires a multidisciplinary approach with first-line medical treatment including ulcer detection, glycemic control and antibiotic therapy if necessary [17]. In the present study a PDT therapy was used as a conservative treatment with an excellent outcome [5,12]. PDT, which uses light to generate in-situ reactive oxygen species, causes cell death even in antibiotic-resistance organisms causing foot infections [5,12,21,22]. A resistant infection may lead to prolonged hospitalization and indiscriminate use of new antibiotics that could generate new resistant strains thereby worsening hospital infection rates [23]. In the case of osteomyelitis, a limited surgical resection of the infected bone may be performed and in case of associated PAD a revascularization procedure must precede bone resection [15]. If blood flow to the extremities is insufficient, it impairs delivery of antibiotics and of oxygen. Therefore, PAD is a very important factor to predict the outcome of the diabetic foot and revascularization should be performed [24]. Although there are some options to improve microcirculation in diabetic patients, we would like to mention the recent result published by Trignano and co-workers that showed an impressive improvement in the level of transcutaneous oxygen in diabetic patients from 29.1±5.4 mmHg to 45.8±6.4 mmHg after operative tarsal tunnel release [25].

It had already been established that PDT, as a conservative treatment, avoided the need for amputation in more than 80% of the cases of diabetic patients who presented foot complications and osteomyelitis [5,12,21]. The Tardivo score has been useful method to direct actions in the treatments by the PDT protocol, but this score shall also be useful to direct actions of any other salvation protocol in diabetic patients with foot complications. Any help in the decision to initiate conservative treatments instead of progressing to amputation can reduce public health costs.

The proposed score classification system for the diabetic foot may enable better quality of life for diabetic patients and promote better low-cost care for millions of individuals worldwide. The adoption of this score associated with antimicrobial photodynamic therapy could reduce amputations in diabetics in over 80% of cases, resulting in lower costs, fewer hospitalizations and no side effects.

## Conclusions

Patients with diabetic foot need to be treated using a multidisciplinary approach. Diabetes does not have cure and the risks to patients may persist throughout life. The Tardivo algorithm is a fundamental tool for predicting whether the diabetic foot has a higher chance of healing or a higher chance of requiring amputation and may be a useful for guiding treatment.

The proposed score classification system for the diabetic foot may enable better quality of life for diabetic patients and promote better low-cost care for millions of individuals worldwide. The adoption of this score associated with antimicrobial photodynamic therapy could reduce amputations in diabetics in over 80% of cases, resulting in lower costs, fewer hospitalizations and no side effects.
